# Response of methanogenic community and their activity to temperature rise in alpine swamp meadow at different water level of the permafrost wetland on Qinghai-Tibet Plateau

**DOI:** 10.3389/fmicb.2023.1181658

**Published:** 2023-05-05

**Authors:** Hongpeng Cui, Yanfa Wang, Xin Su, Shiping Wei, Shouji Pang, Youhai Zhu, Shuai Zhang, Chenjie Ma, Weiguo Hou, Hongchen Jiang

**Affiliations:** ^1^Key Laboratory of Marine Mineral Resources and Polar Geology, Ministry of Education, China University of Geosciences, Beijing, China; ^2^State Key Laboratory of Biogeology and Environmental Geology, China University of Geosciences, Beijing, China; ^3^School of Ocean Sciences, China University of Geosciences, Beijing, China; ^4^Oil and Gas Survey, China Geological Survey, Beijing, China

**Keywords:** Qinghai-Tibet Plateau, permafrost wetland, alpine swamp meadow, methane production, methanogen

## Abstract

Wetlands are an important source of atmospheric methane (CH_4_) and are sensitive to global climate change. Alpine swamp meadows, accounting for ~50% of the natural wetlands on the Qinghai-Tibet Plateau, were considered one of the most important ecosystems. Methanogens are important functional microbes that perform the methane producing process. However, the response of methanogenic community and the main pathways of CH_4_ production to temperature rise remains unknown in alpine swamp meadow at different water level in permafrost wetlands. In this study, we investigated the response of soil CH_4_ production and the shift of methanogenic community to temperature rise in the alpine swamp meadow soil samples with different water levels collected from the Qinghai-Tibet Plateau through anaerobic incubation at 5°C, 15°C and 25°C. The results showed that the CH_4_ contents increased with increasing incubation temperature, and were 5–10 times higher at the high water level sites (GHM1 and GHM2) than that at the low water level site (GHM3). For the high water level sites (GHM1 and GHM2), the change of incubation temperatures had little effect on the methanogenic community structure. *Methanotrichaceae* (32.44–65.46%), *Methanobacteriaceae* (19.30–58.86%) and *Methanosarcinaceae* (3.22–21.24%) were the dominant methanogen groups, with the abundance of *Methanotrichaceae* and *Methanosarcinaceae* having a significant positive correlation with CH_4_ production (*p* < 0.01). For the low water level site (GHM3), the methanogenic community structure changed greatly at 25°C. The *Methanobacteriaceae* (59.65–77.33%) was the dominant methanogen group at 5°C and 15°C; In contrast, the *Methanosarcinaceae* (69.29%) dominated at 25°C, and its abundance showed a significant positive correlation with CH_4_ production (*p* < 0.05). Collectively, these findings enhance the understanding of methanogenic community structures and CH_4_ production in permafrost wetlands with different water levels during the warming process.

## Introduction

Methane (CH_4_) is a greenhouse gas that has a significant impact on global climate change (Caldwell et al., 2008). Although CH_4_ only accounts for 1.8 mL/m^3^ in the atmosphere, its greenhouse effect is about 26 times that of CO_2_, contributing 15% to global warming (Dlugokencky et al., 2009; Wang et al., 2014). Wetlands are important ecosystems, and have a high potential to affect the climate system (Kadykalo and Findlay, 2016; Li T. T. et al., 2022). Wetland CH_4_ emissions are the largest natural source in the global CH_4_ budget, and they may play an increasingly important role in atmospheric CH_4_ growth in the future (Zhang Z. et al., 2017). Permafrost is quite vulnerable to temperature rise, and is undergoing significant degradation due to climate warming (Dlugokencky et al., 2009; Wang et al., 2014). Therefore, the CH_4_ emission processes are strongly associated with climate warming and carbon cycling in permafrost wetlands.

Methanogens are the major communities involved in CH_4_ production. The process of methanogenesis is the terminal step in the decomposition of organic matter by microorganisms in anaerobic environments (Thauer, 1998). Biogenic CH_4_ accounts for 74% of all global CH_4_ emissions (Lowe, 2006). CH_4_ is usually produced by acetate- and H_2_/CO_2_-dependent methanogenesis (Cui et al., 2015). Permafrost wetland soils usually contain highly diverse methanogens such as the potentially acetoclastic families *Methanosarcinaceae* and *Methanotrichaceae* (formerly *Methanosaetaceae*) as well as the hydrogenotrophic orders *Methanomicrobiales*, *Methanobacteriales* and *Methanocellales* (formerly *Rice cluster I*; Cui et al., 2018; Wei et al., 2018; Liu et al., 2019). *Methanosarcinales* and *Methanomicrobiales* are the dominant methanogens in the Zoige wetlands on the Tibetan Plateau, where the acetoclastic pathway is the dominant methanogenic pathway of CH_4_ emissions (Zhang et al., 2008; Cui et al., 2015). Meanwhile, *Methanocellales* and *Methanomassiliicoccales* were found in the alpine wetlands of the permafrost region on the Qilian Mountain (Cui et al., 2018; Wei et al., 2018). Moreover, the amount of CH_4_ generated by H_2_/CO_2_ in peat accounts for 50–100% of the total CH_4_ output (Fey et al., 2001, 2004). Many factors will affect CH_4_ emissions from wetlands, such as temperature, water conditions and vegetation biomass (Huttunen et al., 2003; Lowe, 2006; Zhang et al., 2019; Li W. et al., 2022; Wu et al., 2022). Previous studies have shown that CH_4_ emissions from wetlands are more temperature dependent, and that CH_4_ emissions fluxes are higher at higher temperature (Høj et al., 2008; Cui et al., 2015; Liu et al., 2019). However, soil moisture can affect the availability of oxygen, the diffusion rate of gas, and the activity of microorganisms, so that water-saturated soils tended to release CH_4_ (Christensen et al., 1997; Li W. et al., 2022). Thus, CH_4_ production potential varied significantly under different water levels (Kettunen et al., 1999; Huttunen et al., 2003; Wu et al., 2021).

Qinghai-Tibet Plateau is the largest and highest plateau on Earth, with widely distributed permafrost wetlands and alpine swamp meadows, and has been a methane source (Bubier et al., 1995; Chen et al., 2022). Permafrost wetlands play a vital role in terrestrial carbon storage on the Qinghai-Tibet Plateau (Fey et al., 2001; Li X. Q. et al., 2022), and wetlands have the highest CH_4_ emission rates (0.7 mg CH_4_-C m^−2^ h^−1^) due to the water-logged conditions (Chen et al., 2022). Alpine swamp meadows, with 4.9 × 10^4^ km^2^ (~50%) of the natural wetlands on the Qinghai-Tibet Plateau, are typically soil-nutrient-rich and water-logged, and were considered one of the most important ecosystems on the Qinghai-Tibet Plateau (Zhao et al., 2005; Bai et al., 2019; Wei et al., 2022). Previous studies investigated the diversity of methanogens in the permafrost wetland soil samples from the Qinghai-Tibet Plateau or their community changes in response to temperature rise based on gene clone libraries (Cui et al., 2018; Wei et al., 2018). However, there are few incubation studies on the influence of warmer temperatures on soil CH_4_ emissions and microbial communities at the different water conditions in the alpine swamp meadow wetlands on the Qinghai**-**Tibet Plateau, and the main pathways of CH_4_ production and the influencing factors remain unclear. Therefore, in this study, continuous anaerobic incubations at 5°C, 15°C and 25°C temperatures were conducted on soil samples with various water levels in this area by means of simulation experiments. The composition and abundance of methanogenic communities in the incubated wetland soils were analyzed by high-throughput gene sequencing and quantitative polymerase chain reaction (qPCR), and the effects of the dominant methanogenic community on CH_4_ production were further investigated.

## Materials and methods

### Site description

Our experimental sites are located in the permafrost wetlands of gas hydrate area of Qilian Mountain on the northeastern edge of the Qinghai-Tibet Plateau in southwestern China (Li X. Q. et al., 2022) (Supplementary Figure S1), belonging to the natural ecosystem of alpine swamp meadow (Supplementary Figure S2A). Carbon isotope was applied to show that δ^13^CH_4_ in the surface soil of this area ranges from −84.11‰ to −39.81‰, indicating that CH_4_ is produced by microorganisms (Zhang F. G. et al., 2017). From June to August, surface soil temperatures in this area are between 4.7°C to 21.0°C. Precipitation and glacial melting in this area mainly occurs in summer, forming a natural wetland ecosystem of alpine swamp meadow (Wei et al., 2018). Soil samples from the wetland (collected in June 2017) were provided by the Oil and Gas Survey, China Geological Survey.

### Field measurement and sample collection

The atmospheric and the soil surface temperatures were 6.2°C and 5.5°C, respectively, with 4,060 m as the altitude of the sampling points. According to the differences of water level (the depth of the aquifer), three sampling sites were collected and labeled with GHM1 (Supplementary Figure S2B), GHM2 (Supplementary Figure S2C) and GHM3 (Supplementary Figure S2D). Samples were collected by digging into a depth of 0 to 10 cm below the water level. The soil samples were placed into 50 mL centrifuge tubes and sterilized plastic bags. The samples were transported in a box with ice and then stored at 4°C until further use for the experiments (Jiang et al., 2010; Wagner et al., 2013).

### Establishment of incubation experiments

The incubation procedure was adopted from previous studies (Keller et al., 2004; Cui et al., 2015). The soil samples were mixed with anoxic sterile water in a 1:1 (v/v) ratio and then homogenized well. Approximately 20 mL of the mixture was placed in 100 mL sterile test bottles. The bottles were sealed with butyl rubber stoppers and aluminum caps, and flushed with N_2_ to remove the headspace O_2_. According to the change of surface soil temperatures, three incubation temperatures were set at 5°C, 15°C and 25°C and three parallel slurries were made for each temperature. The bottles were continuously incubated in the dark for 12 weeks, and CH_4_ concentrations measured at weeks 1, 2, 4, 6, 8, 10, and 12.

### Geochemistry analysis

The coordinates of the sampling points, altitude, and the depth of water level were measured during the samples collecting. Total organic carbon (TOC), pH values, and moisture were measured by the method adopted from a previous study (Cui et al., 2018), and soil bulk densities were determined using method adopted from Juottonen et al. (2012). The concentrations of CH_4_ in the headspace was measured by gas chromatography using the GC 7890A (Agilent Technologies, United States; Cui et al., 2018).

### DNA extraction

Total genomic DNA was extracted using the FastDNA SPIN kit (MP Biomedicals, United States) for soil by following the manufacturer’s instructions. The DNA extracted from three parallel samples was mixed in equal amounts to form the DNA of each sample.

### QPCR of *mcrA* gene

The abundance of *mcrA* gene was determined using an ABI Prism 7,500 qPCR system (Applied Biosystems, United States). The plasmid of methanogen was used as the template DNA to calculate the number of copies; 10 times dilution was carried out to form a standard solution with six concentration gradients of 10^4^ to 10^9^ copies/μL, which served as a standard curve for gene fluorescence quantitative analysis (*R*^2^ ≥ 0.99). The qPCR primers were ME3MF and ME2r (Nunoura et al., 2008). The total volume of qPCR reaction was 20 μL containing 10 μL of SYBR Premix Ex Taq (TaKaRa, Japan), 10 ng template DNA, 10 pM each of the forward and reverse primers, 10 pM ROX Reference Dye II and the appropriate volume of ddH_2_O. The qPCR amplification consisted of 1 cycle of 95°C for 30 s, followed by 40 cycles of 95°C for 5 s, 60°C for 34 s (Cui et al., 2018).

### PCR amplification and Illumina sequencing

*mcrA* gene of methanogens were PCR amplified using the primers *mcrAF* and *mcrAR* from the DNA incubation samples at week 0 (original soils) and week 12 (Luton et al., 2002). The total volume of PCR reaction was 50 μL, including 5 μL Taq buffer, 0.2 mM dNTPs, 1.5 mM MgCl_2_, 0.25 μM of each primer, 5 U Taq DNA polymerase (Invitrogen, United States) and 20 ng template DNA. PCR amplification conditions: 4 min at 95°C, 30 cycles of 1 min at 94°C, 1 min at 55°C, and 2 min at 72°C, followed by a final extension step of 10 min at 72°C (Cui et al., 2018; Wei et al., 2018). The *mcrA* gene amplicons were visualized on 2% agarose gels followed by cutting the gel and purifying it with the AxyPrep DNA Gel Extraction Kit according to the manufacturer’s instructions (Axygen Biosciences, USA). We performed *mcrA* genes sequencing analysis at week 0 and week 12.

### Statistical analysis

The sequencing was carried out on an Illumina Miseq PE300 platform (Shanghai, China). *mcrA* gene reads were processed using QIIME 2 package (Callahan et al., 2016). OTUs (Operational Taxonomic Units) were defined at the cutoff value of 14.3% for the *mcrA* gene reads (Barbier et al., 2012; Wei et al., 2018). The analysis process of sequencing data was adopted from Cui et al. (2019). The *mcrA* OTU data were searched against the Functional Gene Pipeline/Repository database, and sequences were verified in the National Center for Biotechnology Information (NCBI) non-redundant database. Principal Component Analysis (PCA) was used to explain the results by using CANACO for Windows 4.5. The Pearson correlation of methanogenic community compositions and CH_4_ concentrations was performed using SPSS 22. Program Mothur was used to calculate the Chao 1, Shannon and Simpson diversity indices.

The estimated absolute abundance (EAA) was defined as the product of relative abundance and total number of microbial cells (Dannemiller et al., 2014; Zhang Z. J. et al., 2017). The relative abundance of methanogens was obtained by Illumina sequencing of *mcrA* gene, while the total number of methanogen cells was quantified by quantitative polymerase chain reaction (qPCR).

### Nucleotide sequence accession numbers

The raw reads have been deposited in the NCBI Sequence Read Archive database under accession number of SRP166224.

## Results

### Soil characteristics of different sampling sites

The physical and chemical characteristics of three sampling sites are shown in Table 1. The three sampling sites had the same altitude. Water level and moisture are higher at site GHM1 (9.20 ± 1.06 cm and 67.28 ± 1.61%), and are lower at site GHM3 (0.13 ± 0.02 cm and 44.74 ± 0.66%). The pH of the soil samples ranged from 6.45 ± 0.02 to 6.76 ± 0.07, belonging to slightly acid soil; the pH of GHM1 was the lowest among the three sampling sites. There was no significant difference in the TOC content of the soil from the three sampling sites.

**Table 1:** Physical and chemical characteristics of the three sampling sites in this experiment.

Site	Soil parameters						
	Location	Altitude (m)	Water level (cm)	Moisture (%)	Bulk density (g/cm^3^)	pH	TOC (g/Kg)
GHM1	38°05′31.39″N 99°10′28.48″E	4,067	9.20 ± 1.06	67.28 ± 1.61	0.68 ± 0.005	6.45 ± 0.02	62.75 ± 0.32
GHM2	38°05′31.18″N 99°10′28.74″E	4,068	3.00 ± 1.32	56.06 ± 1.53	0.71 ± 0.019	6.76 ± 0.07	77.83 ± 1.99
GHM3	38°05′30.92″N 99°10′29.00″E	4,068	0.13 ± 0.02	44.74 ± 0.66	0.80 ± 0.032	6.66 ± 0.10	61.39 ± 1.22

### Change regulation of CH_4_ production

**Figure 1:**
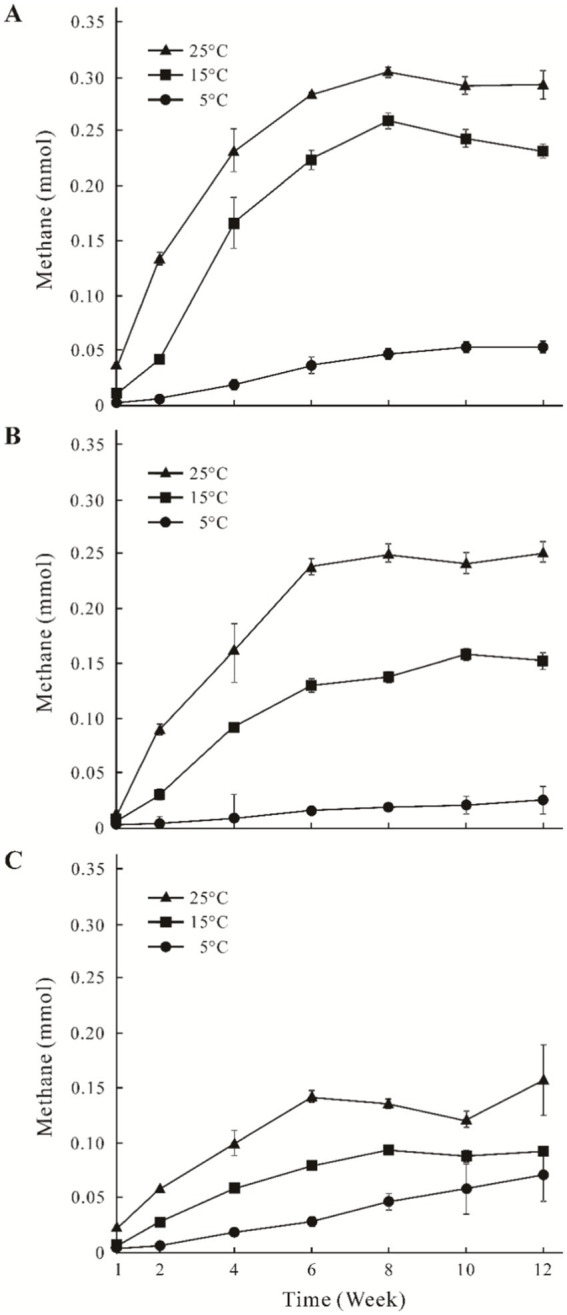
Time course accumulation of CH_4_ in the incubation microcosms at different temperatures using alpine swamp meadow samples. Panels **(A–C)** indicate CH_4_ accumulation of sample GHM1, GHM2, and GHM3, respectively.

The CH_4_ accumulation of the three sites increased from 0.01 mmol to 0.31 mmol with the higher incubation temperature (Figure 1). In general, time-course accumulation of CH_4_ was divided into two phases (the first 6 weeks, and the second 6–12 weeks). CH_4_ production was higher in the first phase than that in the second phase, and the concentrations of CH_4_ remained stable. Concentrations of CH_4_ in the GHM1 treatments at 5°C, 15°C and 25°C peaked at 0.05 mmol, 0.25 mmol and 0.31 mmol, respectively (Figure 1A). Concentrations of CH_4_ in the GHM2 treatment at 5°C, 15°C, and 25°C peaked at 0.06 mmol, 0.16 mmol and 0.25 mmol, respectively (Figure 1B). Concentrations of CH_4_ in the GHM3 treatments at 5°C, 15°C, and 25°C peaked at 0.06 mmol, 0.08 mmol and 0.15 mmol, respectively (Figure 1C).

### Abundance of *mcrA* gene

**Figure 2:**
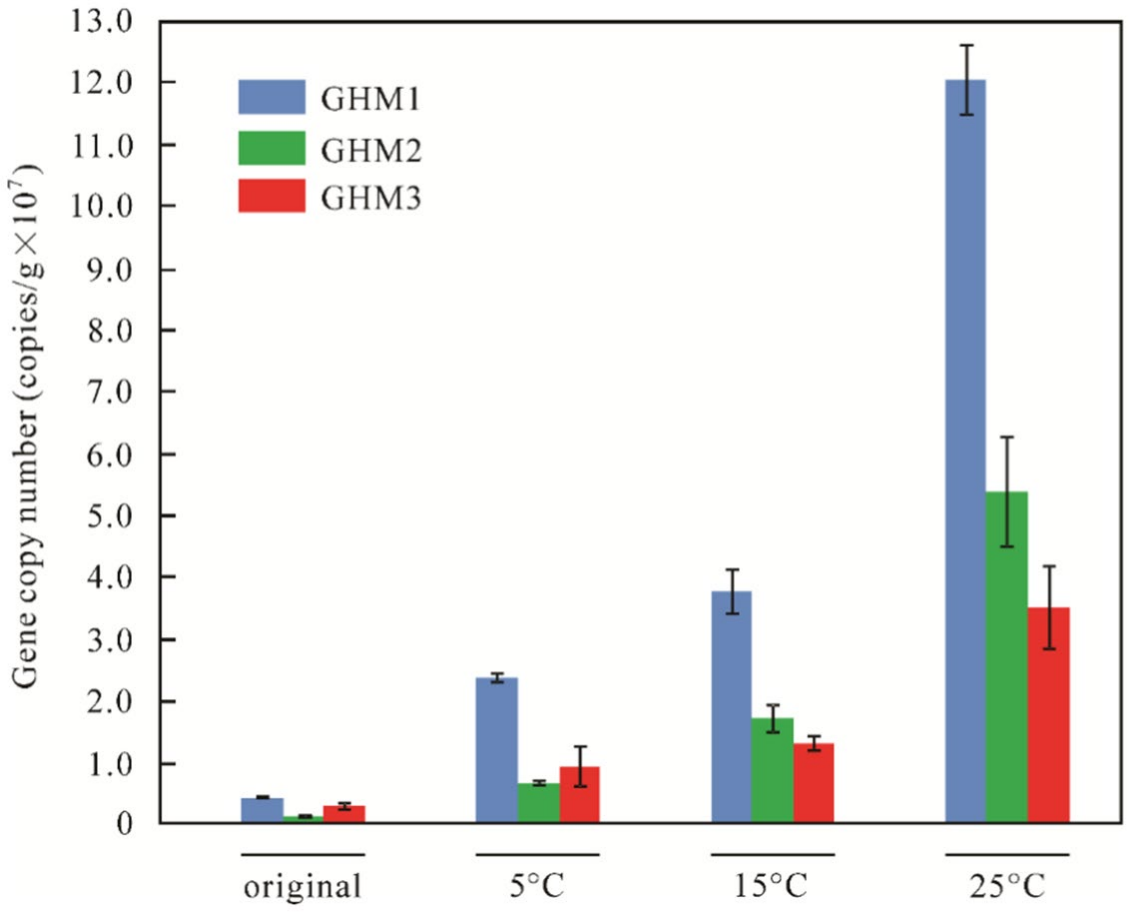
qPCR-based *mcrA* gene abundance in the studied samples at different temperature.

Results of qPCR revealed that the abundance of *mcrA* genes in three original soil samples was 3.85 ± 0.23 × 10^6^ copies/g soil, 1.06 ± 0.29 × 10^6^ copies/g soil, and 2.53 ± 0.52 × 10^6^ copies/g soil, respectively. The abundance of *mcrA* genes changed significantly with increasing incubation temperatures (Figure 2). With the production of biogenic CH_4_ under incubation conditions, the abundance of *mcrA* gene copies of GHM1 was 6–31 times that of the original sample, while it was 5–48 times for GHM2 and 3–13 times for GHM3. The abundance of *mcrA* genes in GHM2 increased the most at 25°C, reaching 5.20 ± 0.87 × 10^7^ copies/g soil. The *mcrA* gene abundance in GHM3 was the least at 5°C, only 3.5 times that of the original sample. The GHM1 sample had the highest abundance of *mcrA* gene at 25°C, reaching 1.21 ± 0.06 × 10^8^ copies/g soil.

### Sequencing and alpha (α) diversity indices

We conducted Illumina sequencing analysis of the *mcrA* gene of 12 samples from three types of incubations. The number of clean reads was 447,148 with 37,262 average number of reads and an average length of 421 bp. The number of OTUs of the 12 samples ranged between 245 to 377, and the coverage was nearly 0.99. The Chao 1, Simpson, and Shannon diversity indices varied 712.29–1778.98, 0.04–0.13, and 3.27–4.48, respectively (Table 2). The temperature rise had less influence on the diversity of the methanogens of GHM1 and GHM2. However, the diversity of methanogens of GHM3 decreased at 25°C compared to the other two incubation temperatures.

**Table 2:** Sequencing information and statistical analyses of *mcrA* gene sequencing of the studied samples.

Samples	No. of Reads	No. of OTUs	Chao 1	Shannon	Simpson
GHM1_01	37,356	290	1095.70	3.70	0.08
GHM1_05	38,552	326	1426.13	3.67	0.11
GHM1_15	35,366	361	1571.59	4.14	0.06
GHM1_25	32,193	245	1069.89	3.46	0.12
GHM2_01	37,434	357	1599.14	4.28	0.04
GHM2_05	39,144	372	1778.98	4.32	0.05
GHM2_15	34,347	358	1701.10	4.48	0.04
GHM2_25	40,916	366	1471.06	4.32	0.05
GHM3_01	49,491	377	1616.08	4.12	0.05
GHM3_05	26,943	289	1383.53	4.27	0.05
GHM3_15	40,482	321	1420.89	3.70	0.09
GHM3_25	34,924	251	712.29	3.27	0.13

### Methanogen diversity based on *mcrA* gene

**Figure 3:**
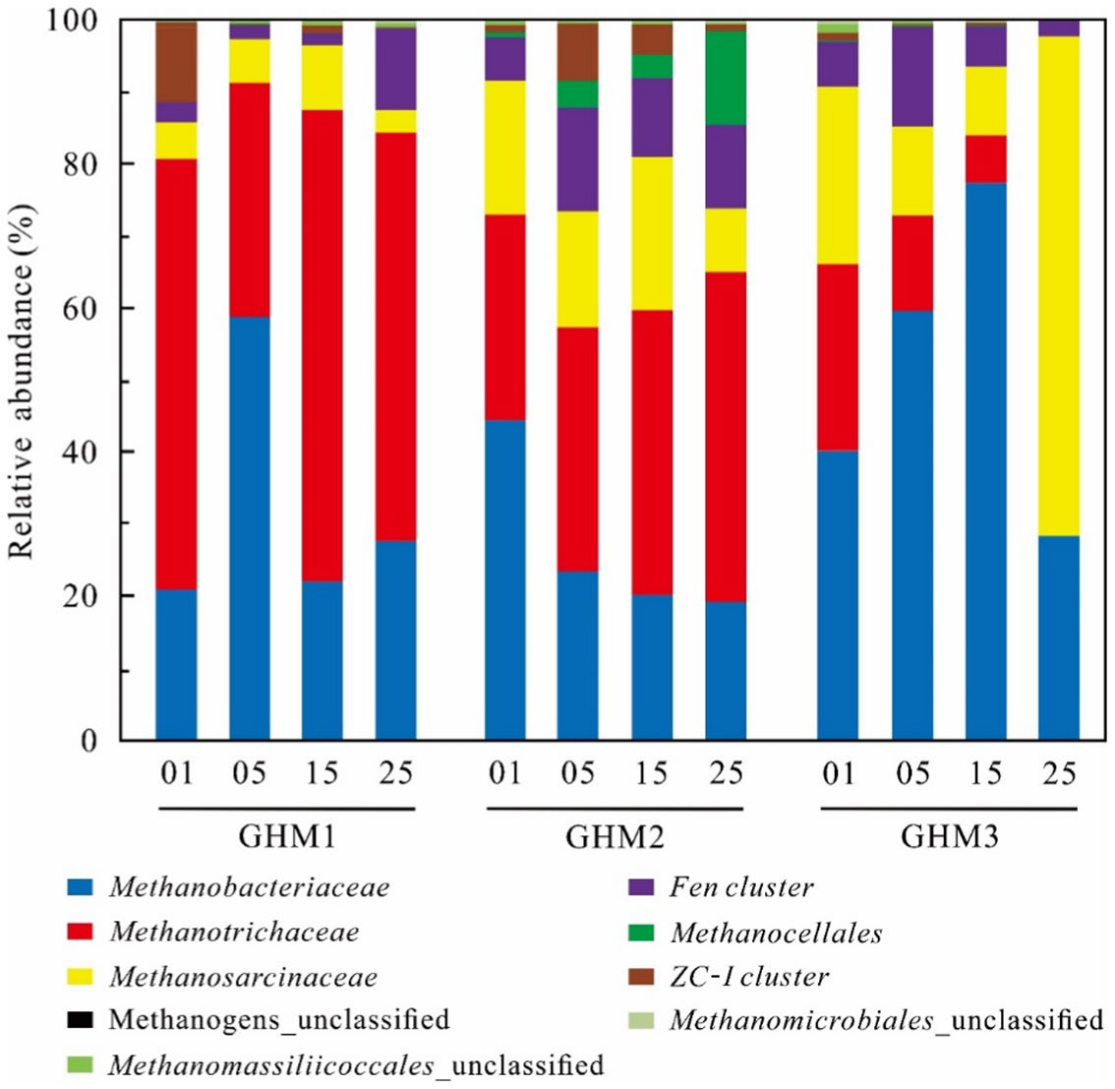
Methanogenic community composition in the studied samples at different incubation temperature. “01” indicates the original soils; “05,” “15,” and “25” indicates the samples incubated at 5°C, 15°C, and 25°C, respectively.

The dominant methanogen group of the GHM1 sample in the original soil was *Methanotrichaceae*, which accounted for 59.81% of the total sequences, followed by *Methanobacteriaceae* (20.95%), *ZC-I cluster* (10.44%), and *Methanosarcinaceae* (4.99%; Figure 3). The *Methanotrichaceae* (32.44–65.46%), *Methanobacteriaceae* (22.05–58.86%) and *Methanosarcinaceae* (3.22–9.00%) were the dominant groups at the incubation temperatures. With increasing temperature, the relative abundance of *Fen cluster* increased at 25°C (11.39%). For sample GHM2, *Methanotrichaceae* (28.64%), *Methanobacteriaceae* (44.41%), and *Methanosarcinaceae* (18.58%) were the dominant methanogen groups, followed by *Fen cluster* (5.94%) in the original soil. The relative abundance of *Methanobacteriaceae*, *Methanosarcinaceae* and *ZC-I cluster* decreased with the increase of incubation temperature, ranging 19.30–23.51%, 8.93–21.24% and 0.93–7.91% in the incubations at 5°C, 15°C and 25°C, respectively. *Methanotrichaceae* and *Methanocellales* increased gradually from 33.90 to 45.68%, and from 3.33 to 12.87%, respectively. For sample GHM3, *Methanotrichaceae* (25.82%), *Methanobacteriaceae* (40.22%), and *Methanosarcinaceae* (24.70%) were the dominant methanogen groups, followed by *Fen cluster* (6.41%) in the original soil. The relative abundance of *Methanobacteriaceae* increased from 59.65 to 77.33%, while that of *Methanotrichaceae* and *Methanosarcinaceae* decreased at 5°C and 15°C from 13.24 to 6.60% and from 12.38 to 9.57%, respectively. *Methanosarcinaceae* became the dominant methanogen group at 25°C, accounting for 69.29% of the total sequences.

**Figure 4:**
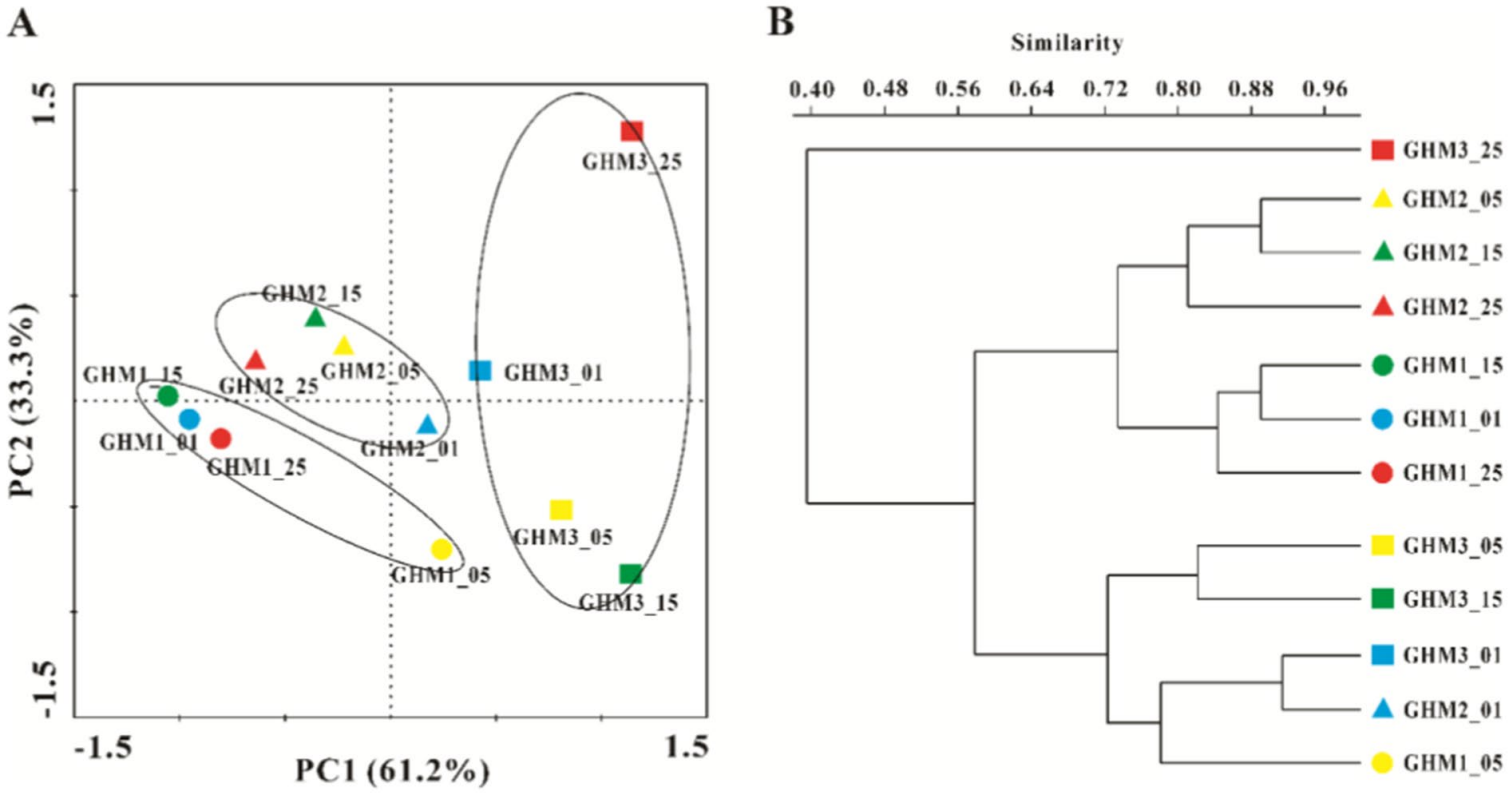
Differences in methanogenic communities structure of all the studied samples. Panel **A**: PCA analysis of methanogenic communities structure of all the studied samples; Panel **B**: Cluster analysis of methanogenic communities structure based on Bray–Curtis similarity index (Boot *N* = 9,999).

PCA analysis detected differences in the methanogenic community compositional structure in the 12 samples (Figure 4A). After Incubation, the community structure of the methanogens in the GHM1 and GHM2 samples changed slightly, while that of the methanogens in the GHM3 samples changed greatly. Cluster analysis further confirmed that the GHM3 samples incubated at 25°C had lower similarity (40%) than the other samples in terms of community composition of methanogens (Figure 4B).

### Estimated absolute abundance

The estimated absolute abundance (EAA) of the dominant methanogens at different incubation temperatures is displayed in Supplementary Table S1. We further analyzed the correlation between the EAA values of the dominant methanogens and CH_4_ production (Supplementary Table S2). These results indicated that the EAA values of *Methanobacteriaceae*, *Methanotrichaceae*, and *Methanosarcinaceae* were relatively higher at different incubation temperatures than *Fen cluster*, *Methanocellales*, and *ZC-I cluster*. For the GHM1 and GHM2 samples, the EAA values of four methanogen groups, *Methanotrichaceae*, *Methanosarcinaceae*, *Fen cluster*, and *Methanocellales*, increased with increasing incubation temperatures, and the EAA values of *Methanotrichaceae*, *Methanosarcinaceae*, and *Methanocellales* were positively correlated with the CH_4_ production (*p* < 0.01). For the GHM3 sample, the EAA value of *Methanosarcinaceae* increased with increasing incubation temperatures, and there was a significant positive correlation between the EAA value and CH_4_ production (*p* < 0.05). In addition, the EAA values of *Methanotrichaceae* and *Methanocellales* decreased with increasing incubation temperatures, with a significant negative correlation between the EAA value and CH_4_ production (*p* < 0.05).

## Discussion

Temperature and soil moisture are the major factors affecting the CH_4_ emissions. Temperature affects the substrate supply of methanogens, thereby affecting their CH_4_ production rate (Brooks et al., 2003). The CH_4_ production in the studied soil samples was positively correlated with the incubation temperature. Previous studies showed that the production of CH_4_ of incubated wetland samples increased with increasing incubation temperature (Jiang et al., 2010; Cui et al., 2018), and that the CH_4_ production in the alpine wetlands incubated at 15°C was 7 times that of the original sample collected from the Qilian Mountain permafrost on the Qinghai**-**Tibet Plateau (Wei et al., 2018). In this study, CH_4_ accumulations in the GHM1, GHM2, and GHM3 samples increased by 5–6, 6–10, and 1–2 times at 5°C, 15°C, and 25°C, respectively (Figure 1). Methanogens are highly sensitive to soil pH and tend to live in weak acidic environments (Metje and Frenzel, 2005, 2007; Xing et al., 2022). Previous studies have revealed that the slightly acidic (pH 6.35–6.75) and high TOC content in permafrost soil can increase CH_4_ production (Barbier et al., 2012; Wei et al., 2018). In this study, the pH of the three incubation systems ranged 6.45–6.75, belonging slightly acid, and the TOC content was similar between the highest water level site (GHM1) and the lowest site (GHM3). Apparently, the soil moisture might support the CH_4_ emissions, as the concentrations of CH_4_ were higher in high water level sites (GHM1 and GHM2) than that in the low water level site (GHM3).

Generally, *Methanotrichaceae*, *Methanobacteriaceae*, and *Methanosarcinaceae* were the dominant methanogenic groups in the high water level sites (GHM1 and GHM2) at high temperature, while the *Methanobacteriaceae* and *Methanosarcinaceae* were the major methanogenic groups in the low water level site (GHM3). Acetoclastic and hydrogenotrophic methanogenesis are the two main methanogenic pathways in the wetlands on the Qinghai**-**Tibet Plateau (Conrad, 1999; Cui et al., 2018). It was found that acetoclastic methanogenesis was widely distributed across various locations, such as Siberian peat bog, flooded Italian rice fields, Zoige wetlands, high Arctic peat, and anaerobic reactors (Fey et al., 2001; Høj et al., 2005; Metje and Frenzel, 2007; Zhang et al., 2008; Qin et al., 2019). In this study, the dominant methanogenic communities were *Methanotrichaceae*, *Methanobacteriaceae*, and *Methanosarcinaceae*. *Methanotrichaceae* most likely use the acetoclastic pathway, while *Methanobacteriaceae* the hydrogenotrophic pathway (Thauer et al., 2008; Oren, 2014; Liu et al., 2019). *Methanosarcinaceae* can use H_2_, acetate, methanol, and other C1 compounds for methanogenic activity (Liu and Whitman, 2008). After incubation at different temperatures, the composition of methanogenic communities showed little change in the GHM1 and GHM2 samples. The abundance of dominant methanogenic groups *Methanotrichaceae* and *Methanosarcinaceae* were positively correlated with CH_4_ production (*p* < 0.01) in the GHM1 and GHM2 (Supplementary Table S2). It has been suggested that the occurrence of *Methanotrichaceae* was related to soil moisture in wetlands (Cui et al., 2018), and they showed the highest relative abundance in the soil where acetate served as the only substrate (Fey and Conrad, 2000). *Methanotrichaceae* utilized only acetate (Boone and Castenholz, 2001). The relative abundance of *Methanotrichaceae* was higher at 15°C and 25°C of the GHM1 and GHM2 samples, while another dominant methanogenic group *Methanobacteriaceae*, which ranged 22.05–58.86% and 19.30–23.51% in the GHM1 and GHM2, respectively, presented an opposite pattern. *Methanobacteriaceae* can use H_2_ and CO_2_ as substrates for methanogenesis, and previous studies have shown that this group can proliferate under certain hydrogen pressure (Conrad and Klose, 2006; Liu and Whitman, 2008). For the GHM3 sample (low water level), the change in incubation temperatures had a great influence on the methanogenic community structure. In general, the abundance of *Methanobacteriaceae* and *Methanosarcinaceae* was higher in GHM3 than in GHM1 and GHM2, while the abundance of *Methanotrichaceae* showed an opposite trend. *Methanobacteriaceae* (59.65 and 77.33%) was the dominant methanogen group at 5°C and 15°C, while presented a relative low abundance at 25°C. *Methanosarcinaceae* (69.29%) was the most abundant methanogen group at 25°C. The temperature between 15–25°C might be the threshold for methanogenic community change in the GHM3 sample (low water level). Moreover, the abundance of *Methanosarcinaceae* had a significant positive correlation with CH_4_ production (*p* < 0.05), while the abundance of *Methanotrichaceae* showed a contrary pattern (Supplementary Table S2). *Methanosarcinaceae* existed in various wetlands such as peatlands, freshwater marshes, paddy soils and low moisture site, and mainly utilized acetate, various methyl compounds, and hydrogen as methanogenic substrates (Hori et al., 2007; Noll et al., 2010; Liu et al., 2012; Cui et al., 2018).

The minor methanogenic communities, such as *Methanocellales*, *Fen cluster* and *ZC-I cluster* were also detected in the three sites. Moreover, the relative abundance of *Fen cluster* and *Methanocellales* of the GHM1 and GHM2 samples increased with the incubation temperature, and had a significant positive correlation with CH_4_ production (*p* < 0.05; Supplementary Table S2). *Fen cluster* belongs to *Methanomicrobiales* (Conrad et al., 2008), and the relative abundance of *Fen Cluster* was higher in the GHM1 and GHM2 samples incubated at 25°C. The members of *Fen Cluster* were relatively tolerant to a changing water table and low pH (Yrjälä et al., 2011). *Methanocellales* harbored pathways for hydrogenotrophic methanogenesis, and was widely distributed across various locations, such as rice fields, terrestrial as well as marine ecosystems (Krüger et al., 2005; Lu et al., 2005; Sakai et al., 2007; Watanabe et al., 2009). The abundance of *Methanocellales* was higher in the GHM2 samples, and especially higher at 25°C (Supplementary Table S1). Moreover, the abundance of *Methanocellales* exhibited a negative correlation with methane production in the GHM3 samples (*p* < 0.05; Supplementary Table S2). *ZC-I cluster* belonged to *Methanosarcinales* (Conrad et al., 2008), and *ZC-I cluster* was mainly found in the Zoige wetlands with its main metabolic substrate including methanol, trimethylamine, acetate, and H_2_/CO_2_, excluding formate (Zhang et al., 2008). In this study, the abundance of *ZC-I cluster* had no significant positive correlation with CH_4_ production of the three sites.

## Conclusion

Soil moisture and temperature are the main factors affecting CH_4_ production in alpine swamp meadow of the permafrost wetland on the Qinghai-Tibet Plateau. Enhanced soil moisture facilitates the production of methane. Temperature rise has an inhibitory effect on the growth and methanogenic activity of *Methanotrichaceae* and *Methanocellales*. These findings may help us better understand the methane cycle in alpine swamp meadow of the permafrost wetland and its response to climate warming.

## Data availability statement

The datasets presented in this study can be found in online repositories. The names of the repository/repositories and accession number(s) can be found in the article/Supplementary material.

## Author contributions

XS and HJ conceived the work. SP, YZ, and SZ performed on-site measurements and collected the samples. HC, YW, CM, and WH analyzed geochemistry and microbiology of the samples. HC, SW, and YW analyzed the sequencing data. HC, SW, XS, and HJ drafted the manuscript. All authors contributed to the article and approved the submitted version.

## Funding

This research was supported by Funds of Oil and Gas Survey, China Geological Survey (Grant nos. GZH201400308, DD20160222, and DD20160226), the 111 project (no. B20011), and the Fundamental Research Funds for the Central Universities (no. 2-9-2020-030).

## Conflict of interest

The authors declare that the research was conducted in the absence of any commercial or financial relationships that could be construed as a potential conflict of interest.

## Publisher’s note

All claims expressed in this article are solely those of the authors and do not necessarily represent those of their affiliated organizations, or those of the publisher, the editors and the reviewers. Any product that may be evaluated in this article, or claim that may be made by its manufacturer, is not guaranteed or endorsed by the publisher.
