# Supplementary material for: Response of methanogenic community and their activity to temperature rise in alpine swamp meadow at different water level of the permafrost wetland on Qinghai-Tibet Plateau
Source: Front Microbiol. 2023 May 5;14:1181658. doi: 10.3389/fmicb.2023.1181658 (PMC10198574; doi:10.3389/fmicb.2023.1181658)
Supplement: Supplementary file 1 [file Data_Sheet_1.PDF]

Supplementary materials

Fig. S1 Location of the experimental site of the Qinghai-Tibet plateau.

Fig. S2 Landscape overview of the three sampling sites on the Qinghai-Tibet Plateau. (A) the natural ecosystem of alpine swamp meadow; (B), (C) and (D) indicates three sampling points of GHM1, GHM2 and GHM3, respectively.

Table S1. Estimated absolute abundance (EAA) of the dominant methanogens at different temperatures.

Table S2. Correlation analysis between estimated absolute abundance (EAA) and methane production by the dominant methanogens.

Fig. S1

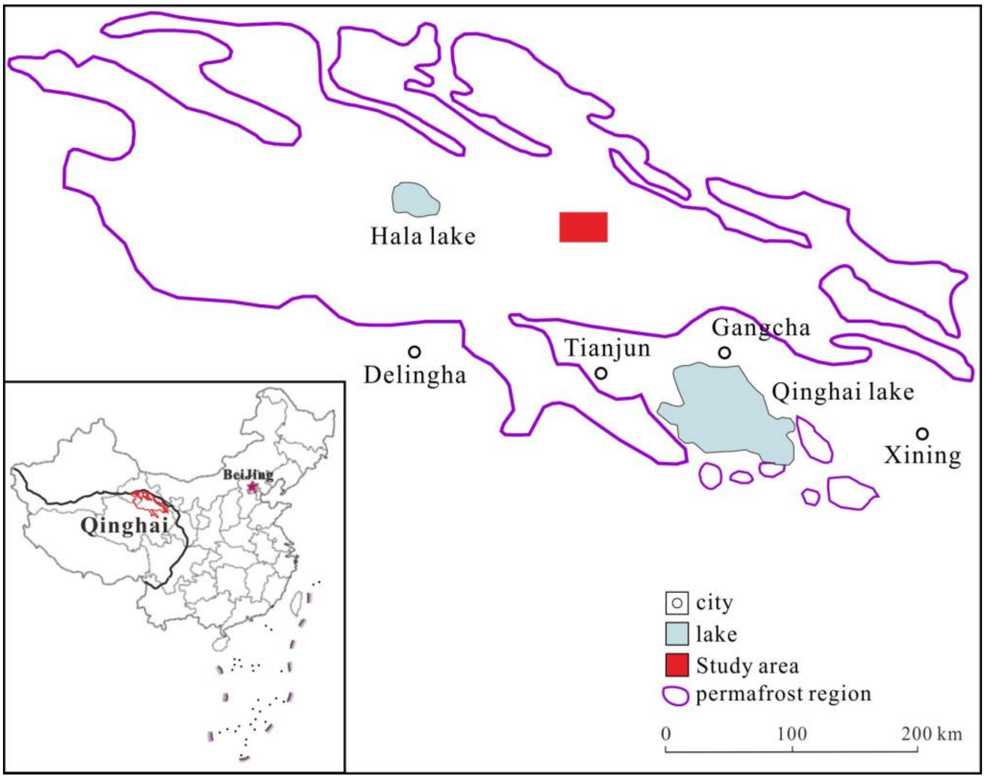

Fig. S1 Location of the experimental site of the Qinghai-Tibet plateau.

Fig. S2

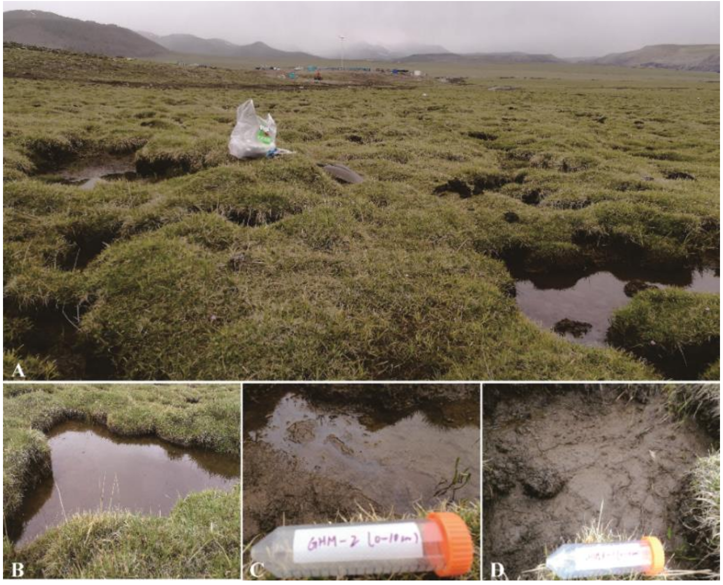

Fig. S2 Landscape overview of the three sampling sites on the Qinghai-Tibet Plateau. (A) the natural ecosystem of alpine swamp meadow; (B), (C) and (D) indicates three sampling points of GHM1, GHM2 and GHM3, respectively.

Table S1. Estimated absolute abundance (EAA) of the dominant methanogens at different temperatures.

Sample	Methanogen	EAA (copies/g soil×10^7)		
		5°C	15°C	25°C
GHM1	*Methanobacteriaceae*	1.28±0.09	0.89±0.06	3.32±0.13
	Methanotrichaceae	0.70±0.04	2.64±0.16	6.80±0.25
	Methanosarcinaceae	0.13±0.009	0.36±0.02	0.39±0.01
	Fen cluster	0.04±0.003	0.07±0.004	1.37±0.05
	Methanocellales	0.0004±0.00003	0.003±0.0002	0.004±0.0001
	ZC-I cluster	0.003±0.0002	0.05±0.003	0.02±0.0008
GHM2	*Methanobacteriaceae*	0.15±0.007	0.33±0.02	0.95±0.06
	Methanotrichaceae	0.21±0.01	0.65±0.04	2.25±0.14
	Methanosarcinaceae	0.10±0.005	0.35±0.02	0.44±0.03
	Fen cluster	0.09±0.004	0.18±0.01	0.58±0.04
	Methanocellales	0.02±0.001	0.06±0.003	0.63±0.04
	ZC-I cluster	0.05±0.002	0.07±0.004	0.05±0.002
GHM3	*Methanobacteriaceae*	0.52±0.12	1.01±0.06	0.99±0.12
	Methanotrichaceae	0.11±0.03	0.09±0.005	0.0006±0.00005
	Methanosarcinaceae	0.10±0.02	0.12±0.008	2.41±0.29
	Fen cluster	0.12±0.03	0.07±0.004	0.08±0.01
	Methanocellales	0.002±0.0005	0.001±0.00008	0
	ZC-I cluster	0.0003±0.00007	0	0

Table S2. Correlation analysis between estimated absolute abundance (EAA) and methane production by the dominant methanogens.

Sample	Methanogen	r	P (2-tailed)
GHM1	*Methanobacteriaceae*	0.599	0.087
	Methanotrichaceae	0.891	0.001**
	Methanosarcinaceae	0.972	$<10^{-4}$**
	Fen cluster	0.722	0.028*
	Methanocellales	0.981	$<10^{-5}$**
	ZC-I cluster	0.608	0.082
GHM2	*Methanobacteriaceae*	0.933	0.0002**
	Methanotrichaceae	0.927	0.0003**
	Methanosarcinaceae	0.972	$<10^{-4}$**
	Fen cluster	0.915	0.001**
	Methanocellales	0.857	0.003**
	ZC-I cluster	-0.091	0.816
GHM3	*Methanobacteriaceae*	0.415	0.267
	Methanotrichaceae	-0.754	0.019*
	Methanosarcinaceae	0.724	0.028*
	Fen cluster	-0.462	0.211
	Methanocellales	-0.746	0.021*
	ZC-I cluster	-0.514	0.157

**Significant correlations (P<0.01); *Significant correlations (P<0.05)
